# Effects of Chemical Ripening With Calcium Carbide on Nutritional Composition, Antioxidant Capacity and Heavy Metal Accumulation in Climacteric Fruits

**DOI:** 10.1155/ijfo/7226409

**Published:** 2026-04-30

**Authors:** Abduljalil Muhammad Mode, Umar Faruk Magaji, Musa Sodiq Babatunde, Abdullahi Dandare, Ibrahim Aliyu Dabai, Shamsudeen Umar Dandare

**Affiliations:** ^1^ Centre for Advanced Science Research and Analytical Services, Usmanu Danfodiyo University, Sokoto, Nigeria, udusok.edu.ng; ^2^ Department of Biochemistry and Molecular Biology, Faculty of Science, Federal University, Birnin Kebbi, Nigeria, fubk.edu.ng; ^3^ Department of Biochemistry and Molecular Biology, Faculty of Chemical and Life Sciences, Usmanu Danfodiyo University, Sokoto, Nigeria, udusok.edu.ng; ^4^ Agri-Environment Branch, Agri-Food and Biosciences Institute, Belfast, UK; ^5^ School of Biological Sciences, Queen′s University Belfast, Belfast, UK, qub.ac.uk

**Keywords:** antioxidants, artificial fruit ripening, food safety, nutritional quality, postharvest handling

## Abstract

Fruits are important dietary sources of nutrients, phytochemicals and antioxidants essential for human health. In many developing countries, calcium carbide (CaC_2_) is widely used as a low‐cost artificial ripening agent despite its associated health risks. This study evaluated the impact of CaC_2_ ripening on the nutritional composition, phytochemical content, antioxidant properties and elemental profile of banana (*Musa* spp.), mango (*Mangifera indica*) and plantain (*Musa paradisiaca*). Fruits were naturally ripened (control) or treated with 10 or 30 g/kg CaC_2_. Standard analytical methods were used to determine proximate composition, phytochemicals, antioxidant activity (DPPH, FRAP and TPC), vitamins, mineral elements and heavy metals. CaC_2_ treatment significantly increased ash content while reducing protein, carbohydrate and lipid levels, with fibre responses varying among fruits. Phytochemical profiles were altered in a dose‐dependent and fruit‐specific manner, including reductions in alkaloids and polyphenols and increases in flavonoids and tannins. Antioxidant vitamins (C and E) decreased significantly (*p* < 0.05), and antioxidant activities were generally suppressed, particularly DPPH radical scavenging capacity. Mineral concentrations increased due to contributions from CaC_2_, while toxic heavy metals (arsenic, lead and cadmium) were present in CaC_2_ and accumulated significantly in treated fruits, with arsenic showing the greatest increase. Collectively, these changes indicate that CaC_2_ not only accelerates ripening but may also promote progression toward overripening, leading to nutrient depletion and biochemical deterioration. This study provides an integrated, multiparameter evaluation of CaC_2_‐induced ripening across multiple climacteric fruits and demonstrates direct transfer of toxic contaminants into edible tissues. The findings highlight significant food safety concerns and underscore the need for stricter regulation, control and the promotion of safer, sustainable ripening alternatives.

## 1. Introduction

Fruits are fundamental components of a healthy diet, providing essential nutrients, phytochemicals and antioxidants that support growth, metabolic function and disease prevention. Inadequate fruit consumption is recognised as a major dietary risk factor for morbidity and mortality from noncommunicable diseases (NCDs) worldwide and particularly in sub‐Saharan Africa, where the burden of NCDs continues to rise [1, 2]. Consequently, fruits are among the world′s most important agricultural products.

Fruit ripening is a natural process regulated by complex physiological, biochemical and molecular events. Ethylene (C_2_H_4_), a plant hormone, plays a central role in initiating and coordinating processes such as increased respiration, chlorophyll degradation, carotenoid biosynthesis, sugar–starch conversion and cell wall softening, which collectively determine fruit texture, colour, aroma and flavour [3, 4]. However, the growing demand for ripe fruits and the need to reduce postharvest losses have led to widespread use of artificial ripening agents to accelerate these processes and improve marketability [5].

Commonly used artificial ripening agents include calcium carbide (CaC_2_), ethephon (2‐chloroethylphosphonic acid), methyl jasmonate (MeJA) and C_2_H_4_. Among these, only C_2_H_4_ and MeJA are generally considered safe if used under controlled conditions of temperature and humidity [6]. In contrast, CaC_2_ is widely used in many developing countries due to its low cost and easy accessibility, despite being banned in several regions because of its associated health risks. CaC_2_ is primarily intended for industrial applications such as welding, steel desulfurization and acetylene synthesis [5]. When applied to fruits, it releases acetylene gas, which mimics C_2_H_4_ activity and hastens ripening. However, commercial CaC_2_ often contains toxic impurities such as arsenic (As), cadmium (Cd), lead (Pb) and phosphine, which are hazardous to human health [7, 8].

Consumption of CaC_2_‐ripened fruits has been associated with a wide range of adverse health outcomes, including gastrointestinal irritation, neurological complications, hypoxia, skin damage, kidney failure and increased cancer risk due to the release of carcinogenic by‐products such as arsine and phosphine gases [6, 7, 9]. In addition to direct toxicity, CaC_2_ ripening alters the biochemical and nutritional profile of fruits, often reducing essential macronutrients and antioxidants, while introducing heavy metals and other contaminants [9, 10]. Fruits ripened with CaC_2_ have been reported to be less palatable, nutritionally inferior and potentially unsafe for human consumption [7].

Despite these concerns, CaC_2_ use remains widespread in Nigeria and other developing countries, driven by limited enforcement of food safety regulations, inadequate consumer awareness and the high cost of safer ripening agents such as C_2_H_4_ and MeJA [11, 12]. Although regulatory agencies such as the National Agency for Food and Drug Administration and Control (NAFDAC) have listed CaC_2_ as a restricted chemical in Nigeria, enforcement gaps have allowed its continued use in fruit markets [13].

Given the potential economic and public health consequences of CaC_2_ use, there is a need for a comprehensive evaluation of its effects on fruit quality and safety. This study investigates the impact of CaC_2_ on the proximate composition, phytochemicals, antioxidant properties, mineral elements and heavy metal content in three widely consumed climacteric fruits—banana (*Musa* spp.), mango (*Mangifera indica*) and plantain (*Musa paradisiaca*). By comparing naturally ripened fruits with those treated with two concentrations of CaC_2_, this work reveals fruit‐specific and dose‐dependent effects on both nutritional quality and contaminant accumulation. Unlike previous studies that typically focus on single fruits or a narrow set of parameters, this study adopts an integrated multiparameter approach within a unified experimental framework, enabling a more holistic assessment of ripening‐induced changes. Notably, it demonstrates the direct transfer of toxic heavy metals, particularly As, from CaC_2_ into fruit tissues, accompanied by concurrent nutritional deterioration. This combined evaluation of biochemical degradation and contaminant exposure provides new insights into the dual impact of CaC_2_‐induced ripening on food quality and safety, highlighting the need for safer, more sustainable alternatives.

## 2. Materials and Methods

### 2.1. Study Area and Experimental Design

The study was conducted in Sokoto State, Northwestern Nigeria, which has 23 local government areas and a landmass of 28,232.37 km^2^ (population 3,702,999; 2006 census). The state lies between 11°30 ^′^–13°50 ^′^E and 4°–6°N.

Three widely consumed fruits, banana (*Musa* spp.), mango (*Mangifera indica*) and plantain (*Musa paradisiaca*), representing commonly cultivated Nigerian cultivars such as Agbagba, Alabameji, PITA‐type plantains and mango cultivars like Julie and Ogbomosho [14], were sourced from Kasuwar Daji Market in Sokoto Metropolis. Fruits were randomly selected to minimise bias, ensuring uniformity in size, ripeness and absence of disease. Botanical authentication was carried out at the Herbarium, in the Department of Plant Sciences, Usmanu Danfodiyo University Sokoto (UDUS), with voucher numbers UDUH/ANS/0120 (banana), UDUH/ANS/0758 (mango) and UDUH/ANS/0121 (plantain).

Banana and plantain (*Musa* spp.) cultivated in Nigeria include common plantain cultivars such as Agbagba and Alabameji and improved hybrids like PITA types, which are widely grown for their adaptability, yield and food value [15]. Mango (*Mangifera indica*) production in Nigeria involves several locally adapted cultivars, including Julie, Ogbomosho, Peter and John Bull, valued for their fruit quality and suitability to tropical conditions [14].

The fruits were grouped into three (3):

• Group I: naturally ripened fruits (control).
• Group II: fruits ripened with 10 g/kg CaC_2_ of fruits.
• Group III: fruits ripened with 30 g/kg CaC_2_ of fruits.

Mature, physiologically developed but unripe fruits of banana, mango and plantain were obtained from a local source and transported to the laboratory under ambient conditions. Fruits were selected at the green stage of ripeness to ensure uniformity prior to treatment. Each fruit used for this study was sampled from the same bunch and source, believed to have been cultivated from the same farm, in order to minimise environmental variation. The fruits were purchased and processed within 24 h of acquisition to reduce postharvest variability. Upon arrival at the laboratory, the fruits were sorted to remove damaged or diseased samples, washed with clean water to remove surface contaminants and air‐dried at room temperature before treatment. The experiment followed a completely randomised design. For each fruit type, five (5) fruits were assigned to each treatment group, representing biological replicates. All analytical measurements were performed in triplicate.

**Figure 1:**
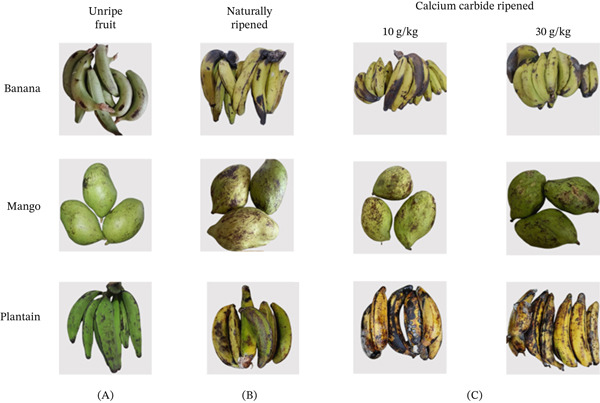
Representative images showing ripeness determination of banana, mango and plantain based on peel colour changes. Columns represent (A) unripe fruits, (B) naturally ripened fruits (control) and (C) calcium carbide (CaC_2_)–ripened fruits. Peel colour change from green to yellow was used as the criterion for ripeness prior to analysis.

For artificial ripening (Groups II and III), freshly harvested unripe fruits were cleaned, weighed (1 kg each) and placed in sealed polyethylene bags inside airtight plastic containers. All samples, including controls, were stored under identical conditions in a closed cupboard for 7–9 days at a room temperature of 30°C–35°C and a relative humidity of 52%–57%. These conditions reflect typical storage environments used by local fruit vendors and were kept consistent across treatments to minimise variation arising from external factors. Ripening progression was monitored daily through changes in peel colour and firmness. The number of days required for each fruit to reach full ripeness was recorded as an indicator of ripening rate under different treatments (Table S1). Fruit ripeness was determined based on peel colour change from green to yellow, as described by Liu [16]. All biochemical analyses were performed when fruits reached a comparable visual ripening stage to minimise variation due to differences in ripening stage. This approach ensured that compositional differences were assessed at equivalent physiological stages rather than at fixed time points. Although detailed ripening kinetics (e.g., C_2_H_4_ production and respiration rate) were not quantitatively measured, the combined use of visual ripeness indicators and ripening duration provided a consistent basis for comparing treatment effects. Representative images illustrating the ripening stages used for sample classification are shown in Figure 1.

Each sample was then divided into two equal portions, each weighing 500 g. One portion was homogenised without any solvent, bottled, labelled and refrigerated for immediate analyses, while the other portion was oven‐dried, pulverised and stored for subsequent assays.

### 2.2. CaC_2_ Collection

CaC_2_ is commercially available in different purity grades, typically ranging from technical grade (approximately 70%–80% CaC_2_) to higher‐purity industrial grades (≥ 90% CaC_2_), depending on the intended application [17]. Technical‐grade CaC_2_, commonly used for industrial purposes such as welding and metal processing, often contains impurities including As and phosphorus compounds that can generate toxic by‐products upon contact with moisture. Although higher‐purity grades may contain fewer impurities, the use of CaC_2_ for fruit ripening remains associated with potential health risks, and its application in food systems is not recommended [7].

In this study, industrial‐grade CaC_2_ was obtained from a welding shop in Sokoto, which represents the type commonly used by local fruit vendors. The compound was applied at concentrations of 10 and 30 g/kg fruit, selected based on previous studies [18] and to reflect common practices in fruit markets. Lower concentrations (10 g/kg) are typically used to induce gradual ripening while maintaining shelf life, whereas higher concentrations (30 g/kg) are used to achieve rapid ripening for immediate sale [19]. These treatment levels enabled evaluation of dose‐dependent effects on ripening behaviour, nutritional composition, phytochemical characteristics, antioxidant capacity and heavy metal accumulation. While the concentrations are not evenly spaced, they were deliberately selected to represent commonly used low/moderate and high application levels in real‐world fruit ripening practices. The objective of this study was therefore not to establish a full dose–response relationship but to evaluate the comparative effects of typical usage scenarios relevant to local fruit markets.

### 2.3. Sample Digestion and Elemental Analysis

Mineral elements (Ca, Cu, Fe, Mg and Zn) and heavy metals (As, Cd and Pb) were quantified using the wet digestion protocol of Magaji et al. [20] Briefly, 0.5 mL of homogenised fruit sample was digested with nitric acid (HNO_3_) and perchloric acid (HClO_4_) in a fume cupboard. After cooling, the digests were diluted with deionised water, filtered and analysed by atomic absorption spectrophotometry (AAS) (PerkinElmer PinAAcle 900H, United States).

### 2.4. Proximate Analysis

The proximate components of each fruit sample were determined using the protocol of the Association of Official Analytical Chemists (AOAC) [21]. Moisture and ash contents were measured by standard oven‐drying and muffle‐furnace procedures, respectively. The Kjeldahl method was used to quantify crude protein, with 6.25 used as the nitrogen‐to‐protein conversion factor. The crude lipid was extracted using petroleum ether by Soxhlet extraction, while crude fibre was quantified using sequential acid and alkali digestion, followed by ashing. Finally, the formula below was used to calculate the carbohydrate content:

Carbohydrate %=100−%moisture+%ash+%protein+%lipid+%fibre.

All determinations were carried out in triplicate, and the results are expressed as percentages on a dry weight basis.

### 2.5. Phytochemical Analysis

Quantitative analyses of the fruit samples were performed to determine the contents of flavonoids, alkaloids, glycosides, saponins, tannins and polyphenols using established protocols. The Bohm and Koupai–Abyazani method was used to determine total *flavonoids* [22]. The *alkaloids* were quantified using the Harborne method by gravimetry [23]. Approximately 5 g of each sample was mixed with 200 mL of 10% acetic acid in ethanol to extract alkaloids. The extract was filtered, and the resulting filtrate was treated with concentrated NH_4_OH to induce alkaloid precipitation. The precipitate was separated, dried at 60°C for 30 min and then weighed. Alkaloid content was calculated as a percentage of the original sample weight.

*Saponins* were also quantified by a modified Harborne method [23]. Briefly, 2 g of each sample was extracted sequentially using diethyl ether and methanol for 3 h each, using a Soxhlet apparatus. The methanol was distilled, while the residues were weighed following drying at 50°C in the oven and then cooling in a desiccator. The saponin content was expressed as a percentage of the original sample weight.

The Folin–Denis spectrophotometric method [24] was used to determine tannins. This was achieved with slight modifications as described by Mode et al. [25]. The pigments were first removed by treating the samples with a solution containing diethyl ether and 1% acetic acid. Tannins were extracted using 70% aqueous acetone for 2 h, followed by filtration, and the extracts were kept at 4°C until further analysis. The reaction mixture was prepared by combining the extracts with Folin–Denis reagent and saturated Na_2_CO_3_ solution and then incubated for 90 min at room temperature. The absorbance was then read at 620 nm, using tannic acid as the reference standard, and tannin concentration was determined as follows:

Tannin mg100g=absorbance of sample×concentration of standardabsorbance of the standard.

*Glycosides* were quantified by the alkaline picrate method [26]. Two millilitres of extract was mixed with 4 mL of alkaline picrate. A blank solution containing 1 mL of distilled water and 5 mL of alkaline picrate reagent was prepared. The reaction mixture was then heated in a water bath for 5 min to allow the formation of a reddish‐brown colour, after which the absorbance was measured at 490 nm. The glycoside concentration was determined using the following formula:

Glycoside mg100g=absorbance of sample×40170×sample size,

where 40 and 170 represent the dilution and gradient factors, respectively.

*Polyphenols* were quantified as described by Singleton et al. [27]. Approximately 0.1 g of the extract was dissolved in 100 mL of distilled water, and 1 mL of this solution was transferred into a test tube. Subsequently, 0.5 mL of 2 N Folin–Ciocalteu reagent was added and gently mixed, followed by 1.5 mL of 20% Na_2_CO_3_ solution. The mixture was brought to a final volume of 8 mL with distilled water, thoroughly mixed and allowed to stand for 2 h. The absorbance was then recorded at 765 nm, and total polyphenol content was determined using a gallic acid calibration curve.

### 2.6. Antioxidant Vitamins

Antioxidant vitamins (C and E) were quantified using colourimetric methods adapted from Mode et al. [25].

For *vitamin C*, 0.5 g of each sample was homogenised in 10 mL of 0.4% oxalic acid and allowed to stand for 10 min. The mixture was then centrifuged at 2000 rpm for 5 min, and the resulting supernatant was filtered. One millilitre of the filtrate was reacted with 9 mL of 2,6‐dichlorophenol indophenol reagent, and the absorbance was recorded at 520 nm using ascorbic acid as the calibration standard. Vitamin C concentration (mg/dL) was calculated using the following formula:

Vitamin C mgdL=absorbance of sample×concentration of standardabsorbance of the standard.

For *vitamin E*, 0.5 g of each sample was extracted with 20 mL of *n*‐hexane, macerated and centrifuged. The solvent layer was evaporated to dryness, and the residue was saponified by heating with 2 mL of 0.5 M alcoholic KOH for 30 min, followed by cooling. Vitamin E was re‐extracted using 3 mL of *n*‐hexane, evaporated and dissolved in a mixture of 2 mL of ethanol, 1 mL of 0.2% ferric chloride and 1 mL of 0.5% *α*,*α*‐dipyridyl. The absorbance was measured at 520 nm against a blank. *α*‐Tocopherol served as the standard, and vitamin E concentration (mg/dL) was determined as

Vitamin E mgdL=absorbance of sample×concnentration of standardabsorbance of the standard.

### 2.7. Antioxidant Activity

The antioxidant activity was evaluated using three assays: 2,2‐diphenyl‐1‐picrylhydrazyl (DPPH) radical scavenging capacity, ferric reducing antioxidant power (FRAP) and total phenolic content (TPC). Methods described by Alyaqoubi et al. [28] and Dandare et al. [29] were followed, with methanol used as the extraction solvent for all assays. Approximately 100 g of each fruit sample was extracted with 500 mL of methanol and filtered, and the filtrate was concentrated using a rotary evaporator maintained at 45°C–60°C. The resulting extract was reconstituted to prepare five concentrations (0.2, 0.4, 0.6, 0.8 and 1.0 mg/mL), which were used for all antioxidant assays.

For the DPPH radical scavenging test, both the stock and working solutions were prepared. The stock solution was obtained by dissolving 40 mg DPPH in 100 mL of methanol and stored at −20°C until use. To prepare the working solution, equal volumes of the stock solution and methanol were combined, and the absorbance was adjusted to approximately 1.0 ± 0.01 at 517 nm. For analysis, 1 mL of the working DPPH solution was mixed with 100 *μ*L of each extract concentration and incubated for 2 h in the dark. The radical scavenging activity was then evaluated based on the reduction in absorbance measured at 517 nm.

For the FRAP assay, the reagent was freshly prepared by mixing 300 mM acetate buffer (pH 3.6), 10 mM TPTZ dissolved in 40 mM HCl and 20 mM FeCl_3_·6H_2_O in a 10:1:1 volumetric ratio. A 100 *μ*L portion of each extract was mixed with 1 mL of the FRAP reagent and incubated for 30 min at room temperature. The absorbance of the reaction was then recorded at 595 nm. Antioxidant capacity was quantified using a Trolox calibration curve, and the results were expressed as milligrammes of Trolox equivalents per 100 g dry weight (mg TE/100 g DW).

The TPCs were determined using a modified Folin–Ciocalteu procedure. In brief, 100 *μ*L of the extract was combined with 0.4 mL of distilled water and 0.5 mL of diluted Folin–Ciocalteu reagent, followed by incubation for 5 min at ambient temperature. Subsequently, 1 mL of 7.5% sodium carbonate (*w*/*v*) was added, and the mixture was allowed to stand for 2 h. Absorbance was measured at 765 nm, and TPC was calculated from a gallic acid standard curve, expressed as milligrammes of gallic acid equivalents per 100 g dry weight (mg GAE/100 g DW).

### 2.8. Statistical Analysis

All data are presented as mean ± standard deviation (SD) of triplicate determinations. Statistical analyses were performed using GraphPad Prism Version 10.6.1 (San Diego, United States). Differences among groups were evaluated by one‐way analysis of variance (ANOVA) followed by Tukey′s multiple comparison post hoc test. Statistical significance was accepted at *p* < 0.05.

## 3. Results and Discussion

The effects of CaC_2_ ripening on the nutritional and chemical composition of banana, mango and plantain are presented below. Changes in proximate composition, phytochemicals, antioxidant properties, mineral elements and heavy metal concentrations were evaluated by comparing naturally ripened fruits with those treated with two concentrations of CaC_2_. It should be noted that detailed ripening kinetics (e.g., C_2_H_4_ production and respiration rate) were not quantitatively measured in this study. However, fruits were analysed at a comparable visual ripening stage, and ripening duration was recorded, providing a consistent basis for interpreting treatment effects. Therefore, the observed compositional differences are attributed primarily to CaC_2_ treatment, although potential variations in ripening dynamics cannot be entirely excluded.

### 3.1. Proximate Composition of CaC_2_‐Ripened Fruits

**Figure 2:**
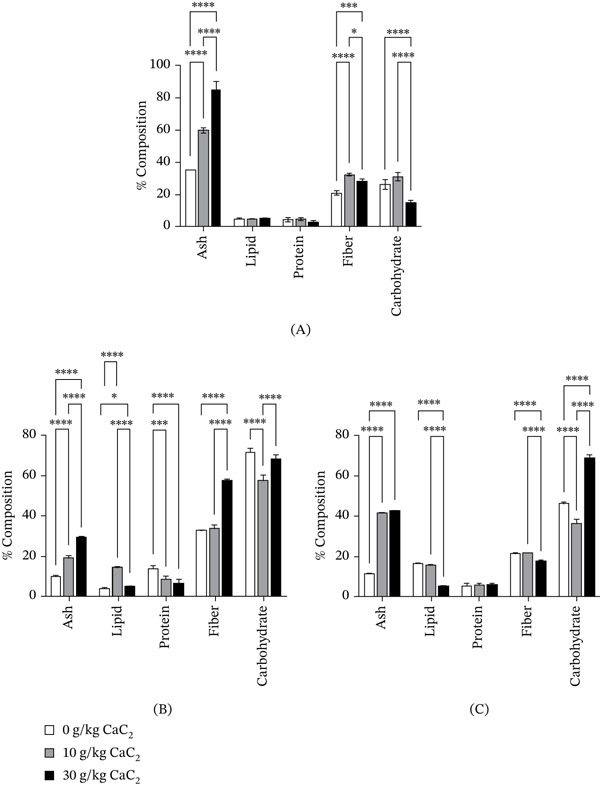
Percentage proximate composition on a dry weight basis for (A) banana, (B) mango and (C) plantain ripened with and without calcium carbide. Bars represent the mean, and error bars represent the standard deviation (*n* = 3). Asterisks above the bars indicate statistical significance between treatments; absence of asterisks denotes no significant difference.

The proximate composition (on a dry weight basis) of banana, mango and plantain ripened with and without CaC_2_ is shown in Figure 2. CaC_2_ treatment significantly altered the nutrient composition in all fruits, with concentration‐ and fruit‐specific effects.

Ash content, an indicator of total mineral concentration [30], has increased significantly in all CaC_2_‐ripened fruits compared with naturally ripened controls. The highest increase was observed in banana at 30 g/kg CaC_2_ fruit (19.85%), compared with 10.96% in the control. Similar trends were recorded for mango (2.88%–5.96%) and plantain (2.96%–9.94%). These increases likely reflect that CaC_2_ contributes exogenous minerals during ripening, consistent with earlier reports in mango and pawpaw [18]. However, Chinagorom et al. [31] observed reductions, suggesting that fruit type and ripening conditions may influence mineral dynamics. The higher ash content in CaC_2_‐ripened fruits may result from mineral residues such as calcium hydroxide formed when CaC_2_ reacts with moisture, which can deposit on or diffuse into fruit tissues and increase inorganic material detected during ash analysis. In addition, acetylene‐induced accelerated ripening may enhance moisture loss and concentrate minerals within tissues, thereby increasing relative ash percentage compared with naturally ripened fruits [8, 18].

Carbohydrate levels generally declined with CaC_2_ treatment. Banana fell from 8.10% in the control to 3.45% at 30 g CaC_2_, while mango dropped from 20.46% to 13.76%. Plantain decreased at 10 g CaC_2_ (11.7%–8.72%) but rose again at 30 g CaC_2_ (14.15%). These reductions are consistent with accelerated starch hydrolysis driven by acetylene gas, which acts as a C_2_H_4_ analogue and stimulates amylase activity [32]. Prolonged ripening may also deplete sugars through enhanced respiration. The partial carbohydrate recovery in plantain at higher CaC_2_ concentration may reflect varietal differences in starch metabolism or preharvest maturity [33]. Similar reductions have been reported in CaC_2_‐ripened bananas [31, 34]. The decline in carbohydrate content in CaC_2_‐treated fruits is consistent with acetylene‐induced acceleration of ripening, as acetylene mimics C_2_H_4_ and stimulates respiration and enzymatic breakdown of stored carbohydrates, leading to rapid utilisation during ripening [35]. The increase observed in plantain at higher CaC_2_ concentration may reflect extensive starch hydrolysis into soluble sugars at more advanced ripening stages [36].

Fibre content varied among fruits. Mango fibre increased at 30 g CaC_2_ (11.66% vs. 9.34% in control), whereas plantain fibre declined steadily (5.50%–3.65%), and banana showed an inconsistent pattern. Fibre breakdown during ripening is typically mediated by enzymes such as cellulase and pectinase [37]. The increase in mango may suggest delayed enzymatic activity or differences in lignin metabolism [38]. This contrasts with Adeyemi et al. [18], who reported consistent fibre reductions after CaC_2_ ripening, again pointing to species‐specific metabolic responses.

Lipid content also showed fruit‐dependent changes. Banana (1.48%–1.22%) and plantain (4.29%–1.10%) decreased under CaC_2_ treatment, whereas mango increased sharply at 10 g CaC_2_ (3.80% vs. 1.13% in control) before declining at higher concentrations. Lipids, though minor fruit constituents, serve as precursors for aroma volatiles that contribute to fruit flavour [39]. The observed lipid increase in mango may reflect enhanced mobilisation for volatile synthesis, consistent with findings in climacteric fruits [40]. Differences between fruits may reflect species‐specific metabolism or ripening conditions.

Moisture content increased significantly across all fruits following CaC_2_ treatment, reaching 76.6% in banana, 79.9% in mango and 79.5% in plantain at 30 g CaC_2_ compared with 68%–75% in controls (Table S1). This increase, although expressed on a fresh weight basis, is consistent with fruit tissue softening and osmotic changes induced by acetylene [9]. Elevated moisture is associated with reduced shelf life and increased microbial spoilage [32], explaining the poor storability commonly reported for chemically ripened fruits.

Protein content decreased significantly in all CaC_2_‐treated fruits, with the greatest reduction observed in banana (1.32%–0.58%). Protein breakdown during ripening is linked to proteolytic activity and mobilisation of amino acids for respiration [41]. CaC_2_, through acetylene release, may accelerate this proteolysis, consistent with reports of enhanced protein degradation in mango and pawpaw ripened with CaC_2_ [18, 42].

CaC_2_‐induced ripening of fruits caused consistent reductions in protein, carbohydrate and lipid contents while increasing ash and moisture levels, with fibre responses being fruit‐specific. These alterations indicate significant biochemical disruptions that compromise fruit nutritional quality and reduce shelf stability. Collectively, the observed reductions in carbohydrate, protein, lipid and antioxidant components, alongside increased moisture content, suggest that CaC_2_ treatment may not only accelerate ripening but also promote progression towards overripening or early senescence. In climacteric fruits, rapid exposure to C_2_H_4_ or C_2_H_4_ analogues can intensify respiration and metabolic turnover, leading to accelerated depletion of sugars, degradation of cellular constituents and tissue softening beyond optimal ripeness. The pattern observed at the higher CaC_2_ dose (30 g/kg) supports this interpretation, indicating that strong C_2_H_4_‐like stimulation may advance ripening beyond the normal physiological optimum. This is consistent with the known effects of chemically induced ripening, which can enhance starch hydrolysis, increase respiration, accelerate softening, promote moisture accumulation and deplete antioxidants [9, 32]. Consequently, the lower carbohydrate, protein, lipid, vitamin C and TPC values observed at higher CaC_2_ concentration likely reflect an advanced or overripened physiological state rather than simple ripening progression. Overall, these findings confirm that CaC_2_ ripening undermines both the nutritional value and postharvest integrity of climacteric fruits.

In addition to chemical ripening agents such as CaC_2_, postharvest temperature regimes are known to influence ripening‐associated biochemical and nutritional changes in climacteric fruits. In mango, storage temperature affects carbohydrate metabolism and ripening enzyme activity, with higher temperatures accelerating ripening processes, while low‐temperature storage suppresses respiration and C_2_H_4_ production, thereby delaying quality changes [43, 44]. Similarly, in *Musa* fruits (banana and plantain), postharvest conditions, including temperature, have been shown to influence starch–sugar conversion, oxidative status and antioxidant capacity during ripening [45, 46]. However, as all fruits in the present study were ripened under comparable ambient conditions, the observed differences are primarily attributable to CaC_2_ treatment.

### 3.2. Phytochemical Composition of CaC_2_‐Ripened Fruits

**Figure 3:**
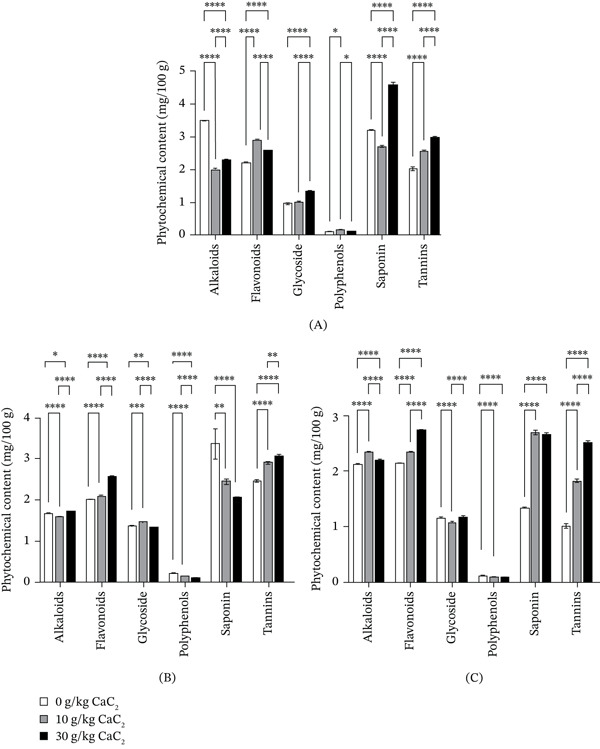
Phytochemical composition for (A) banana, (B) mango and (C) plantain ripened with and without calcium carbide. Bars represent the mean, and error bars represent the standard deviation (*n* = 3). Asterisks above the bars indicate statistical significance between treatments; absence of asterisks denotes no significant difference.

The phytochemical composition of banana, mango and plantain ripened with and without CaC_2_ is presented in Figure 3. Six phytochemical parameters were examined: alkaloids, flavonoids, glycosides, polyphenols, saponins and tannins. CaC_2_ treatment induced significant, fruit‐specific and concentration‐dependent changes, reflecting broad alterations in secondary metabolism.

Alkaloid content generally decreased across all fruits following CaC_2_ ripening. In bananas and mangoes, alkaloid levels dropped markedly compared with controls, with the lowest values recorded in mangoes treated with 10 g CaC_2_ (1.59 mg/100 g). This reduction may result from suppressed biosynthesis or accelerated degradation during chemically induced ripening. Similar declines have been reported in CaC_2_‐ripened mango and banana [47], although Chinagorom et al. [31] documented increased alkaloids in bananas, suggesting that species and ripening conditions influence responses. Flavonoids, well known for their antioxidant and anti‐inflammatory roles, increased consistently in all fruits after CaC_2_ treatment. In bananas, levels rose from 2.21 mg/100 g in controls to 2.88 mg/100 g at 10 g CaC_2_, with comparable increases in mangoes and plantains. This trend aligns with previous reports in CaC_2_‐ripened bananas [31] and is likely linked to stress‐induced biosynthesis triggered by acetylene, which mimics C_2_H_4_ and promotes the accumulation of secondary metabolites [48].

Glycoside responses varied. Significant increases were recorded in banana and mango, whereas plantain showed a decrease at 10 g CaC_2_ but a recovery at 30 g. This pattern indicates that CaC_2_ affects glycoside biosynthetic pathways in a dose‐ and species‐dependent manner. Modest increases in cyanogenic glycosides have also been reported following CaC_2_ treatment in bananas [31]. Polyphenols declined significantly in mango and plantain at higher CaC_2_ concentrations, reaching 0.102 and 0.105 mg/100 g, respectively, at 30 g CaC_2_. In banana, however, polyphenol levels showed a slight increase at 10 g CaC_2_. These findings suggest that oxidative degradation of phenolics occurs under stress or due to differences in the timing of biosynthetic activity. Contrastingly, Chinagorom et al. [31] reported stable polyphenol levels in CaC_2_‐treated fruits, underscoring variability across studies.

Saponins exhibited fruit‐specific responses. Mango showed a steady decline with increasing CaC_2_ (3.35 mg/100 g in control to 2.05 mg/100 g at 30 g). By contrast, plantain saponins increased progressively (1.36–2.72 mg/100 g), while banana showed a dip at 10 g CaC_2_ followed by a sharp rise at 30 g (4.52 mg/100 g). These patterns contrast with reports of reduced saponins in CaC_2_‐ripened bananas [31]. While saponins provide antimicrobial and anticancer benefits, excessive accumulation can present toxicity risks [49]. The decrease in saponins in mango with increasing CaC_2_ may be attributed to acetylene‐induced accelerated ripening, as C_2_H_4_‐like signalling enhances metabolic turnover and degradation of secondary metabolites, including saponins. Conversely, the increase observed in plantain and banana at higher CaC_2_ levels may reflect stress‐induced stimulation of defensive secondary metabolites such as saponins during rapid ripening [35, 36]. Tannin levels increased significantly in all fruits under CaC_2_ treatment. Mango reached the highest concentration at 30 g CaC_2_ (3.04 mg/100 g), while untreated plantain had the lowest (1.03 mg/100 g). These results support earlier findings of elevated tannins in CaC_2_‐treated fruits [31]. Although tannins possess antioxidant and antimicrobial activities, high concentrations may impair nutrient absorption [32]. Tannin levels increased in CaC_2_‐treated fruits, likely due to stress and oxidative signals associated with accelerated ripening, which stimulate the phenylpropanoid pathway and enhance tannin biosynthesis as a protective response [35, 36].

Overall, CaC_2_ ripening reduced alkaloids, polyphenols and, in some cases, saponins, while increasing flavonoids, tannins and glycosides. These biochemical shifts reflect stress‐induced alterations in secondary metabolism, with implications for both the nutritional quality of fruits and potential health risks for consumers.

### 3.3. Antioxidant Properties of CaC_2_‐Ripened Fruits

**Figure 4:**
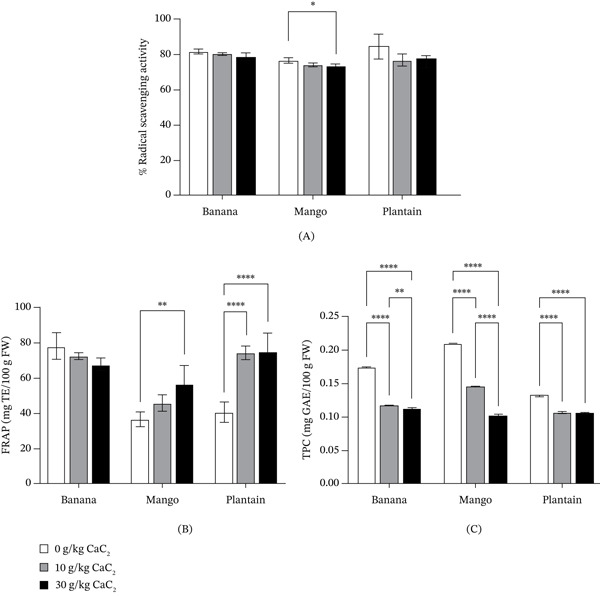
Antioxidant activities (A) 2,2‐diphenyl‐1‐picrylhydrazyl (DPPH) radical scavenging capacity, (B) ferric reducing antioxidant power (FRAP) and (C) total phenolic content (TPC) of fruits ripened with and without calcium carbide. Bars represent the mean, and error bars represent the standard deviation (*n* = 3). Asterisks above the bars indicate statistical significance between treatments; absence of asterisks denotes no significant difference.

The antioxidant vitamins (C and E) and antioxidant activities (DPPH radical scavenging, FRAP and TPC) of banana, mango and plantain ripened with and without CaC_2_ are presented in Table 1 and Figure 4, respectively. CaC_2_ treatment induced marked, fruit‐specific alterations in antioxidant status.

**Table 1:** Antioxidant vitamin concentrations of fruits ripened with and without calcium carbide.

Samples		Vitamin C (mg/100 g)	Vitamin E (mg/100 g)
Banana	0 g/kg CaC_2_	8.874 ± 0.016^a^	0.067 ± 0.004^a^
	10 g/kg CaC_2_	8.407 ± 0.016^b^	0.087 ± 0.004^b^
	30 g/kg CaC_2_	3.609 ± 0.009^c^	0.068 ± 0.002^a^
Mango	0 g/kg CaC_2_	19.573 ± 0.152^a^	0.517 ± 0.004^a^
	10 g/kg CaC_2_	17.695 ± 0.113^b^	0.172 ± 0.007^b^
	30 g/kg CaC_2_	11.542 ± 0.049^c^	0.184 ± 0.003^b^
Plantain	0 g/kg CaC_2_	13.596 ± 0.046^a^	0.068 ± 0.002^a^
	10 g/kg CaC_2_	10.391 ± 0.065^b^	0.048 ± 0.001^b^
	30 g/kg CaC_2_	10.165 ± 0.032^c^	0.042 ± 0.001^c^

*Note:* Data represent mean ± standard deviation of triplicate values. Values with different superscript letters within the same fruit indicate significant differences among treatments at *p* < 0.05.

The application of CaC_2_ during ripening markedly influenced the antioxidant profiles of banana, mango and plantain. A consistent decline in vitamin C across all fruits indicates that chemical ripening accelerates oxidative and thermal degradation processes. This pattern agrees with earlier reports for CaC_2_‐treated bananas, papaya and mango [18, 50]. The exothermic hydrolysis of CaC_2_ releases heat and acetylene gas, both of which can stimulate respiration and oxidative activity, leading to enhanced ascorbic acid breakdown [32]. The effect was particularly pronounced at 30 g/kg CaC_2_, highlighting a dose‐dependent impact on vitamin stability.

Vitamin E responses were more fruit‐specific. Mango exhibited the highest natural tocopherol content but experienced substantial reductions following CaC_2_ exposure, consistent with tocopherol oxidation during accelerated ripening [51]. Plantain showed a similar decline. Interestingly, banana displayed a modest increase in vitamin E at low CaC_2_ concentration, which may reflect a stress‐induced upregulation of tocopherol biosynthesis, as previously observed in climacteric fruits under mild abiotic stress [52]. This suggests that the physiological response to chemical ripening is not uniform across species and may depend on intrinsic antioxidant capacity and ripening physiology.

Our data indicate that CaC_2_ ripening compromises antioxidant vitamin content, especially at higher concentrations, potentially diminishing the nutritional quality of the fruits. The observed fruit‐specific variations underscore the importance of considering crop physiology when evaluating the nutritional effects of chemical ripening practices.

Antioxidant activity assays revealed distinct, fruit‐specific responses to CaC_2_ treatment. DPPH radical scavenging activity showed a numerical decrease in all fruits, indicating a general reduction in free radical–quenching capacity following chemical ripening. However, the decline was not statistically significant in banana and plantain, suggesting the presence of compensatory antioxidant mechanisms. Similar nonsignificant reductions in DPPH activity have been reported in CaC_2_‐treated bananas [53] and may reflect the contribution of other antioxidant compounds that remain stable or increase during ripening stress. CaC_2_‐induced ripening may lower DPPH radical scavenging activity by accelerating metabolism and oxidative stress, which consume nonenzymatic antioxidants; however, banana and plantain may maintain overall capacity through upregulated enzymatic antioxidant defences (glyoxalase–glutathione systems under stress) and stress‐responsive phenolic synthesis [54].

FRAP activity exhibited contrasting trends among the fruits. In banana, FRAP values decreased from 77.62 mg TE/100 g FW in naturally ripened fruits to 66.64 mg TE/100 g FW at 30 g/kg CaC_2_, indicating a reduction in overall reducing power. By contrast, mango and plantain showed substantial increases, rising from 36.18 to 55.89 mg TE/100 g FW and from 39.83 to 74.19 mg TE/100 g FW, respectively. The increase in mango and plantain FRAP suggests that chemical ripening may enhance the availability or formation of nonphenolic reducing compounds or induce shifts in phenolic composition towards more redox‐active constituents. Such patterns have been observed previously in different cultivars of mango [55, 56].

TPC values decreased significantly in all fruits with increasing CaC_2_ concentration. In banana, TPC declined from 0.175 mg GAE/100 g FW in controls to 0.112 mg GAE/100 g FW at 30 g/kg CaC_2_. Mango and plantain exhibited similar trends, with reductions from 0.210 to 0.102 mg GAE/100 g FW and from 0.132 to 0.105 mg GAE/100 g FW, respectively. These decreases are consistent with the oxidative degradation or polymerisation of phenolics during accelerated ripening, as reported by Laryea et al. [53].

Overall, CaC_2_ ripening produced complex, species‐dependent effects on antioxidant activity. While DPPH responses were broadly reduced, FRAP increased in mango and plantain despite declining TPC, indicating that total phenolics alone do not fully account for antioxidant capacity under chemical ripening. These findings highlight how CaC_2_ treatment alters the antioxidant landscape of fruits in a manner that reflects both their intrinsic phytochemical profiles and their physiological responses to ripening stress.

### 3.4. Mineral Element Composition of CaC_2_‐Ripened Fruits

The mineral composition of banana, mango and plantain ripened with and without CaC_2_ is presented in Table 2. CaC_2_ itself contained significantly high calcium (88.99 mg/kg), as well as detectable levels of copper, iron and magnesium, while zinc was below the detection limit. Its mineral content was reflected in the altered profiles of the treated fruits.

**Table 2:** Mineral element composition of fruits ripened with and without calcium carbide.

Samples		Minerals (mg/kg)				
		Ca	Cu	Fe	Mg	Zn
Banana	0 g/kg CaC_2_	3.476 ± 0.056^a^	0.379 ± 0.002^a^	1.074 ± 0.004^a^	1.643 ± 0.008^a^	1.800 ± 0.004^a^
	10 g/kg CaC_2_	18.107 ± 0.049^b^	0.437 ± 0.014^b^	3.514 ± 0.023^b^	2.005 ± 0.008^b^	2.800 ± 0.005^b^
	30 g/kg CaC_2_	5.460 ± 0.082^c^	0.231 ± 0.008^c^	2.191 ± 0.074^c^	1.324 ± 0.008^c^	0.562 ± 0.027^c^
Mango	0 g/kg CaC_2_	3.644 ± 1.333^a^	0.160 ± 0.003^a^	1.091 ± 0.042^a^	0.250 ± 0.021^a^	0.140 ± 0.007^a^
	10 g/kg CaC_2_	15.603 ± 0.025^b^	0.164 ± 0.004^a^	1.116 ± 0.021^a^	0.568 ± 0.020^b^	0.203 ± 0.002^b^
	30 g/kg CaC_2_	11.117 ± 0.021^c^	0.165 ± 0.003^a^	0.593 ± 0.015^b^	0.165 ± 0.003^c^	0.205 ± 0.001^b^
Plantain	0 g/kg CaC_2_	10.827 ± 0.031^a^	0.240 ± 0.008^a^	0.399 ± 0.025^a^	0.606 ± 0.008^a^	0.175 ± 0.003^a^
	10 g/kg CaC_2_	11.427 ± 0.005^b^	0.276 ± 0.005^b^	0.687 ± 0.006^b^	1.042 ± 0.011^b^	0.670 ± 0.002^b^
	30 g/kg CaC_2_	18.840 ± 0.040^c^	0.276 ± 0.005^b^	0.802 ± 0.026^c^	1.310 ± 0.006^c^	1.114 ± 0.006^c^
CaC_2_		88.990 ± 5.536	0.213 ± 0.004	0.344 ± 0.010	0.440 ± 0.008	BDL

*Note:* Data represent mean ± standard deviation of triplicate values. Values with different superscript letters within the same fruit indicate significant differences among treatments at *p* < 0.05. The CaC_2_ row represents the mineral composition of the chemical used for treatment.

Abbreviation: BDL, below detectable limit.

Calcium levels increased significantly in all CaC_2_‐treated fruits. In banana, Ca rose sharply from 3.48 mg/kg in the control to 18.11 mg/kg at 10 g/kg CaC_2_, before declining significantly at 30 g/kg (5.46 mg/kg). Plantain also showed a significant increase, reaching 18.84 mg/kg at 30 g/kg CaC_2_ compared to 10.83 mg/kg in the control. Mango exhibited a significant elevation at 10 g/kg (15.60 mg/kg), followed by a decline at 30 g/kg (11.12 mg/kg), though still higher than the untreated control. Calcium levels in CaC_2_‐treated fruits likely increased due to deposition and absorption of calcium hydroxide formed during CaC_2_ hydrolysis, whereas declines at higher doses may result from saturation of binding sites or leaching, with species differences reflecting tissue permeability and retention [8, 18]. These findings corroborate earlier reports [57, 58] and suggest that calcium from CaC_2_ diffuses into fruit tissues, altering mineral composition through direct leaching or changes in membrane permeability.

Copper content increased significantly in banana (0.38–0.44 mg/kg at 10 g/kg CaC_2_) and plantain (0.24–0.28 mg/kg), while no significant differences were observed in mango across treatments. This observation in banana was also reported by Ubuoh et al. [59]. Such enrichment may be linked to carbide‐induced alterations in membrane integrity that facilitate ion uptake. Copper has also been reported to enhance C_2_H_4_ activity by saturating metal‐binding sites [60], suggesting an indirect role in ripening acceleration.

Iron concentrations varied significantly among fruits and treatment levels. In banana, Fe increased from 1.07 mg/kg in the control to 3.51 mg/kg at 10 g/kg CaC_2_, followed by a significant reduction to 2.19 mg/kg at 30 g/kg. Mango Fe content showed no significant change at 10 g/kg but declined significantly at 30 g/kg (0.59 mg/kg). In contrast, plantain displayed a consistent and significant increase across treatments, rising from 0.40 to 0.80 mg/kg. These patterns align with earlier findings on CaC_2_‐ripened bananas [59], indicating that moderate exposure enhances Fe accumulation, whereas higher doses may impair transport or storage. Iron levels in CaC_2_‐treated fruits may vary due to stress‐induced mobilisation and species‐specific retention, while declines at higher doses may arise from precipitation or binding to cellular ligands that limit extractable Fe [61, 62].

Magnesium and zinc levels were also significantly modulated by CaC_2_ treatment. At 10 g/kg, Mg and Zn increased significantly in all fruits, with banana Mg reaching 2.01 mg/kg and Zn reaching 2.80 mg/kg. At 30 g/kg, banana Mg and Zn both declined significantly (1.32 and 0.56 mg/kg, respectively), while plantain maintained elevated concentrations, and mango showed intermediate responses. Mg and Zn levels in CaC_2_‐treated fruits increased at low doses due to enhanced uptake and mobilisation but declined at higher doses in some fruits, likely from transport saturation or antagonistic mineral interactions, with species differences reflecting tissue and storage characteristics [63]. These results support the interpretation that moderate CaC_2_ exposure promotes mineral uptake, whereas excessive treatment disrupts mineral homeostasis, possibly through interference with enzyme activity or transport processes. It also corroborates previous reports [57, 59].

Ripening by CaC_2_ treatment significantly altered the mineral composition of the fruits, increasing calcium, copper and iron contents while modulating magnesium and zinc in a dose‐dependent manner. Although these changes may suggest improved mineral content, such increases are unlikely to represent true nutritional enhancement. While elevated levels of essential elements such as Ca and Fe may contribute to dietary intake, excessive or imbalanced concentrations of trace elements such as Cu and Zn could pose health risks if consumed beyond recommended limits [7]. In addition, the observed mineral enrichment likely reflects contamination from CaC_2_, which contains impurities, rather than physiological uptake by the fruit. This raises concerns regarding the safety and authenticity of the nutritional profile of artificially ripened fruits. Furthermore, CaC_2_ use is associated with additional toxicological risks, including exposure to acetylene and associated impurities, which have been linked to mucosal irritation and systemic toxicity [64]. Collectively, these findings highlight potential consumer health risks and underscore the need for stricter regulation and safer alternatives to chemical ripening practices.

### 3.5. Heavy Metal Composition of CaC_2_‐Ripened Fruits

The commercial CaC_2_ used in this study contained high levels of As (21.75 mg/kg), Pb (0.88 mg/kg) and Cd (0.05 mg/kg) (Table 3). These findings are consistent with earlier reports that commercial‐grade CaC_2_ contains toxic impurities, including phosphorus hydride, As and heavy metals [7, 65]. Their presence directly implicates CaC_2_ as a source of contamination during artificial ripening. Based on Codex Alimentarius and FAO/WHO guidelines, permissible limits for heavy metals in fruits are generally around 0.1 mg/kg for Pb and 0.05–0.1 mg/kg for Cd, while no universal maximum level is established for As, although values around 0.1 mg/kg are commonly used for comparison.

**Table 3:** Heavy metal composition of fruits ripened with and without calcium carbide.

Samples		Heavy metals (mg/kg)		
		As	Cd	Pb
Banana	0 g/kg CaC_2_	2.337 ± 1.213^a^	0.002 ± 0.001^a^	0.253 ± 0.026^a^
	10 g/kg CaC_2_	15.460 ± 0.717^b^	0.005 ± 0.001^a^	0.259 ± 0.013^a^
	30 g/kg CaC_2_	17.653 ± 1.690^b^	0.004 ± 0.002^a^	0.229 ± 0.039^a^
Mango	0 g/kg CaC_2_	0.957 ± 0.612^a^	0.002 ± 0.001^a^	0.052 ± 0.021^a^
	10 g/kg CaC_2_	3.723 ± 0.340^b^	0.004 ± 0.001^a^	0.183 ± 0.013^b^
	30 g/kg CaC_2_	5.540 ± 1.118^b^	0.006 ± 0.002^b,a^	0.146 ± 0.020^b^
Plantain	0 g/kg CaC_2_	1.640 ± 0.364^a^	0.001 ± 0.001^a^	0.052 ± 0.021^a^
	10 g/kg CaC_2_	5.930 ± 1.176^b^	0.002 ± 0.000^a^	0.143 ± 0.038^a^
	30 g/kg CaC_2_	9.940 ± 2.436^b^	0.003 ± 0.001^a^	0.183 ± 0.011^b,a^
CaC_2_		21.750 ± 2.045	0.052 ± 0.002	0.881 ± 0.025
Accepted safety limits (WHO/FAO/Codex)		0.1 mg/kg	0.05 mg/kg	0.1 mg/kg

*Note:* Data represent mean ± standard deviation of triplicate values. Within each fruit, values with different superscript letters differ significantly, while values sharing the same superscript letter are not significantly different at *p* < 0.05. In cases where two superscripts are shown (e.g., b,a), the mean differs significantly from Group I (b) but not from Group II (a). The CaC_2_ row represents the heavy metal composition of the chemical used for treatment.

*As* concentrations increased significantly in all CaC_2_‐treated fruits. In banana, As rose from 2.34 mg/kg in the control to 15.46 and 17.65 mg/kg at 10 and 30 g CaC_2_, respectively. Similar dose‐dependent increases were observed in mango (0.96–5.54 mg/kg) and plantain (1.64–9.94 mg/kg). These levels are all substantially above the accepted safety limit of 0.1 mg/kg, indicating potential health risks from consuming CaC_2_‐treated fruits. As levels likely increased due to trace As impurities in commercial CaC_2_, which deposit on or diffuse into tissues, with higher doses and species‐specific tissue properties driving the observed accumulation [66]. Such concentrations far exceed typical guidance values and pose serious public health risks, including cancer, neurotoxicity and cardiovascular, liver and kidney damage, with children and pregnant women particularly vulnerable to chronic exposure [67]. These results are consistent with earlier studies and confirm the transfer of As from CaC_2_ into fruit tissues [7, 68]. Chronic dietary exposure to As is linked to carcinogenesis, neurological impairment and systemic toxicity in humans [69].

*Pb* showed fruit‐specific responses. In mango, Pb increased significantly from 0.05 mg/kg in controls to 0.18 mg/kg at 10 g and remained elevated at 30 g CaC_2_ (0.15 mg/kg). In plantain, Pb also rose from 0.05 mg/kg in the control to 0.18 mg/kg at 30 g CaC_2_, with the increase significant relative to the control. In banana, however, Pb levels (0.25–0.26 mg/kg) did not differ significantly across treatments. Notably, the Pb concentrations in mango, plantain and banana exceed the WHO/FAO/Codex‐accepted safety limit of 0.1 mg/kg, suggesting potential health risks from consuming CaC_2_‐treated fruits. Pb levels in CaC_2_‐treated fruits increased in mango and plantain due to Pb impurities and species‐specific tissue uptake, while banana remained unchanged, possibly due to lower absorption or more effective sequestration, with higher doses limited by saturation of binding sites [66]. These findings align with previous reports of Pb enrichment in CaC_2_‐ripened fruits [10]. Both As and Pb are classified as carcinogens by the International Agency for Research on Cancer (IARC) [70], underscoring their toxicological relevance even at low concentrations.

*Cd* levels were generally lower than those of As and Pb. In banana and plantain, small variations (0.002–0.004 mg/kg) were not significant. In mango, however, Cd increased significantly at 30 g CaC_2_ (0.006 mg/kg) compared to the control (0.002 mg/kg). Despite these increases, Cd levels in all fruits remained below the WHO/FAO/Codex‐accepted safety limit of 0.05 mg/kg, indicating that Cd does not pose a significant health risk in the CaC_2_‐treated fruits. Cd levels remained low in CaC_2_‐treated fruits, with mango showing slight accumulation at high doses due to species‐specific tissue uptake, while banana and plantain showed minimal changes [66]. Although present at trace levels, Cd is highly toxic, with no known biological role, and chronic exposure contributes to kidney damage, bone demineralisation and cancer risk [70].

The accumulation of As, Pb and Cd in CaC_2_‐treated fruits confirms that the chemical is the source of contamination. Among these, As exhibited the most alarming and consistent increases. Beyond diminishing nutritional and antioxidant quality, the presence of such heavy metals substantially elevates the toxicological risk of consuming artificially ripened fruits, reinforcing earlier evidence that CaC_2_ use in food processing poses serious public health hazards [7, 68].

Collectively, the results reveal a consistent pattern in CaC_2_‐ripened fruits: loss of essential nutrients and antioxidants alongside the introduction of exogenous minerals and toxic heavy metals. While some parameters, such as flavonoids, tannins and certain minerals, increased under CaC_2_ treatment, these shifts primarily reflect stress responses or contamination rather than genuine nutritional enhancement. The combination of nutrient depletion, altered phytochemical balance and heavy metal accumulation highlights the complex ways in which chemical ripening undermines fruit quality and safety. These findings set the stage for a broader consideration of CaC_2_′s public health implications.

Several safe and economically feasible alternatives to CaC_2_ are available for fruit ripening. Controlled application of C_2_H_4_ gas remains the most widely adopted method, as it mimics natural ripening and is approved by regulatory bodies such as FAO and IFOAM [3]. Ethephon, a C_2_H_4_‐releasing compound, also provides a practical and cost‐effective option for promoting uniform ripening and colour development [64]. In addition, natural approaches such as plant‐derived coatings and extracts (e.g., clove, cinnamon and neem) offer low‐cost and safe alternatives, particularly suitable for small‐scale and local fruit handlers. More advanced approaches, such as MeJA and 1‐methylcyclopropane (1‐MCP), have also been reported [71], although their application may be limited by cost and accessibility in some settings. These methods provide viable options to replace CaC_2_ while maintaining fruit quality and safety.

## 4. Conclusion

This study demonstrated that CaC_2_ treatment significantly alters the nutritional, phytochemical, antioxidant and elemental composition of banana, mango and plantain. Proximate composition analysis revealed increases in ash content and reductions in key macronutrients such as protein, lipid and carbohydrate, with the extent of change varying among fruits and treatment levels. Phytochemical constituents and antioxidant activities were also modulated in a dose‐dependent and fruit‐specific manner, indicating biochemical disruption during artificial ripening. Importantly, CaC_2_ contained high levels of As, Pb and Cd, which were subsequently detected at significantly elevated concentrations in treated fruits, confirming the chemical as a direct source of contamination.

These findings underscore the nutritional and toxicological risks associated with CaC_2_ use for fruit ripening. The apparent ‘nutrient enrichment’ observed for certain elements reflects contamination rather than genuine improvement in quality. Regulatory enforcement to restrict CaC_2_ use, coupled with public awareness campaigns and promotion of safer ripening alternatives, is urgently needed to protect consumer health and ensure food safety.

## Funding

No funding was received for this manuscript.

## Conflicts of Interest

The authors declare no conflicts of interest.

## Supporting information


**Supporting Information** Additional supporting information can be found online in the Supporting Information section. Table S1: Duration of fruit ripening (days) under different calcium carbide treatments. Table S2: Proximate composition of fruits ripened with and without calcium carbide.

## Data Availability

The data that support the findings of this study are available in the supporting information of this article.
