# Supplementary material for: Effects of Chemical Ripening With Calcium Carbide on Nutritional Composition, Antioxidant Capacity and Heavy Metal Accumulation in Climacteric Fruits
Source: Int J Food Sci. 2026 Apr 30;2026:7226409. doi: 10.1155/ijfo/7226409 (PMC13129937; doi:10.1155/ijfo/7226409)
Supplement: Supplementary file 1 — Supporting Information Additional supporting information can be found online in the Supporting Information section. Table S1: Duration of fruit ripening (days) under different calcium carbide treatments. Table S2: Proximate composition of fruits ripened with and without calcium carbide. [file IJFO-2026-7226409-s001.docx]

**Supplementary Material**

**Table S1:** Duration of Fruit Ripening (Days) Under Different Calcium Carbide Treatments

| **Fruit** | **Duration of fruit ripening (in days)** | |
| --- | --- | --- |
|  | **10 g/kg CaC_2_** | **30 g/kg CaC_2_** |
| Banana | 7 | 4 |
| Mango | 9 | 7 |
| Plantain | 8 | 5 |

**Table S2.** Proximate composition of fruits ripened with and without calcium carbide.

| **Samples** | | **Moisture (%)** | **Ash (%)** | **Lipids (%)** | **Proteins (%)** | **Fibre (%)** | **Carbohydrates (%)** |
| --- | --- | --- | --- | --- | --- | --- | --- |
| **Banana** | 0 g/kg CaC2 | 68.810±2.081^a^ | 10.957±0.013^a^ | 1.480±0.010^a^ | 1.316±0.439^a^ | 6.470±0.005^a^ | 8.104±0.432^a^ |
|  | 10 g/kg CaC2 | 74.080±0.627^b^ | 15.487±0.028^b^ | 1.222±0.003^b^ | 1.170±0.258^a^ | 8.360±0.018^b^ | 8.042±0.868^a^ |
|  | 30 g/kg CaC2 | 76.598±1.513^b^ | 19.852±0.018^c^ | 1.185±0.009^c^ | 0.583±0.253^a^ | 6.530±0.017^c^ | 3.448±0.295^b^ |
| **Mango** | 0 g/kg CaC2 | 71.590±0.153^a^ | 2.878±0.033^a^ | 1.130±0.057^a^ | 3.938±0.438^a^ | 9.335±0.005^a^ | 20.464±0.552^a^ |
|  | 10 g/kg CaC2 | 73.923±1.073^b^ | 5.008±0.019^b^ | 3.798±0.025^b^ | 2.188±0.438^b^ | 8.860±0.010^b^ | 15.083±1.333^b^ |
|  | 30 g/kg CaC2 | 79.947±0.093^c^ | 5.957±0.037^c^ | 1.023±0.021^c^ | 1.317±0.438^b^ | 11.658±0.015^c^ | 13.757±0.373^b^ |
| **Plantain** | 0 g/kg CaC2 | 74.555±0.022^a^ | 2.963±0.015^a^ | 4.290±0.005^a^ | 1.467±0.245^a^ | 11.757±0.229^a^ | 11.757±0.229^a^ |
|  | 10 g/kg CaC2 | 76.208±0.049^b^ | 9.940±0.013^b^ | 3.812±0.006^b^ | 1.417±0.118^a^ | 8.722±0.459^b^ | 8.722±0.459^b^ |
|  | 30 g/kg CaC2 | 79.522±0.044^c^ | 8.767±0.018^c^ | 1.105±0.010^c^ | 1.254±0.069^a^ | 14.151±0.337^c^ | 14.151±0.337^c^ |

Data represent the Mean ± Standard deviation of triplicate measurements. Values with different superscript letters within the same fruit indicate significant differences among treatments at *p* < 0.05.
